# Cryptococcal Lung Infection Simulating Metastasis in a Myxoid Liposarcoma Patient: A Case Report

**DOI:** 10.1155/crdi/5538208

**Published:** 2026-05-13

**Authors:** Mariko M. Kawamura, Masatake Matsuoka, Hideki Ujiie, Shin Ariga, Shinichi Nakazato, Kenjiro Kato, Takayuki Hashimoto, Yoshihiro Matsuno, Tatsuya Kato, Norimasa Iwasaki

**Affiliations:** ^1^ Department of Orthopaedic Surgery, Faculty of Medicine and Graduate School of Medicine, Hokkaido University, North 15 West 7 Kita-ku, Sapporo, 060-8638, Hokkaido, Japan, hokudai.ac.jp; ^2^ Department of Orthopaedic Surgery, Keio University School of Medicine, Shinjuku-ku, 160-0016, Tokyo, Japan, tokyo.ac.jp; ^3^ Department of Thoracic Surgery, Hokkaido University Hospital, Sapporo, 060-8648, Hokkaido, Japan, hokudai.ac.jp; ^4^ Department of Medical Oncology, Faculty of Medicine and Graduate School of Medicine, Hokkaido University, Sapporo, 060-8638, Hokkaido, Japan, hokudai.ac.jp; ^5^ Department of Surgical Pathology, Hokkaido University Hospital, Sapporo, 060-8648, Hokkaido, Japan, hokudai.ac.jp; ^6^ Faculty of Medicine, Global Center for Biomedical Science and Engineering, Hokkaido University, North 15 West 7 Kita-ku, Sapporo, 060-8638, Hokkaido, Japan, hokudai.ac.jp

**Keywords:** case report, liposarcoma, myxoid liposarcoma, pulmonary cryptococcosis, sarcoma

## Abstract

**Introduction:**

Myxoid liposarcoma is a soft tissue sarcoma with a high tendency for lung metastasis. Its aggressive nature necessitates accurate diagnosis and treatment planning to improve outcomes.

**Case Report:**

A 31‐year‐old man presented with a three‐year history of an asymptomatic, progressively enlarging mass in his right thigh. Magnetic resonance imaging indicated a soft tissue tumor within the sartorius muscle, and an incisional biopsy suggested myxoid liposarcoma. Staging computed tomography scans revealed two nodules in the right lung, leading to an initial diagnosis of Stage IV myxoid liposarcoma. Given his overall health and the presumed resectability of the lesions, the patient underwent three courses of preoperative chemotherapy with doxorubicin and ifosfamide. Follow‐up imaging showed no change in the size of the tumor or lung nodules. A wide resection of the thigh tumor was performed, including part of the vastus medialis, sartorius, and gracilis muscles. Postoperatively, 65 gray (relative biological effectiveness) of proton radiation therapy was administered. Subsequently, a robot‐assisted thoracoscopic right lower lobectomy and lymph node dissection were performed to resect the lung nodules. Histological examination of the lung lesions revealed necrotizing granulomas with numerous round‐shaped yeasts. The special stains confirmed pulmonary cryptococcosis, ruling out metastasis and revising the tumor staging to Stage Ib. Consequently, postoperative chemotherapy was canceled, and the patient showed no recurrence over 3 years of follow‐up.

**Discussion:**

This case emphasizes the need for histopathological evaluation of lung lesions in sarcoma patients, as not only cryptococcosis but also various fungal infections can mimic metastasis, potentially altering the treatment plan and prognosis.

## 1. Introduction

Myxoid liposarcoma is a subtype of soft tissue sarcoma that typically occurs in the extremities of young to middle‐aged adults [1]. The lungs are a common site of metastasis; a comprehensive review of 1853 patients with myxoid liposarcoma indicated that pulmonary lesions account for approximately 24% of the first metastatic sites [2]. In a recent large‐scale nationwide study involving 11,132 patients with liposarcoma, myxoid liposarcoma accounted for 12.3% of cases, and 5.0% of these patients presented with metastases at the initial diagnosis [3]. While the 3‐year overall survival rate for myxoid liposarcoma is high at 94.2%, the management of advanced disease remains challenging, with the median survival after the diagnosis of metastases reported to be approximately 30 months in a contemporary cohort [3]. However, the actual frequency of concurrent fungal infections, such as pulmonary cryptococcosis, in patients with myxoid liposarcoma remains unestablished due to a lack of systematic data. This case highlights a critical diagnostic pitfall, as such infections can closely mimic metastatic recurrence in this specific oncological context.

The treatment strategy for advanced myxoid liposarcoma, when both the primary and metastatic tumors are resectable, typically involves surgical resection of both the primary and metastatic tumors, possibly combined with adjuvant therapy [4, 5]. In contrast, if the primary and metastatic tumors remain unresectable despite chemotherapy, palliative therapy becomes the appropriate course of treatment. In such cases, pathological analyses of the metastatic lesions are sometimes not performed.

Here, we report a case of myxoid liposarcoma diagnosed as American Joint Committee on Cancer (AJCC) 8^th^ edition [6] Stage IV at initial presentation because of a lung lesion. The patient is young, and all identified lesions were deemed surgically resectable, making them eligible for curative treatment. After adjuvant chemotherapy followed by surgical resection of the primary and “metastatic” lesions, the pathology of the lung specimen revealed that the lung lesions were actually pulmonary cryptococcosis.

## 2. Case Presentation

**FIGURE 1:**
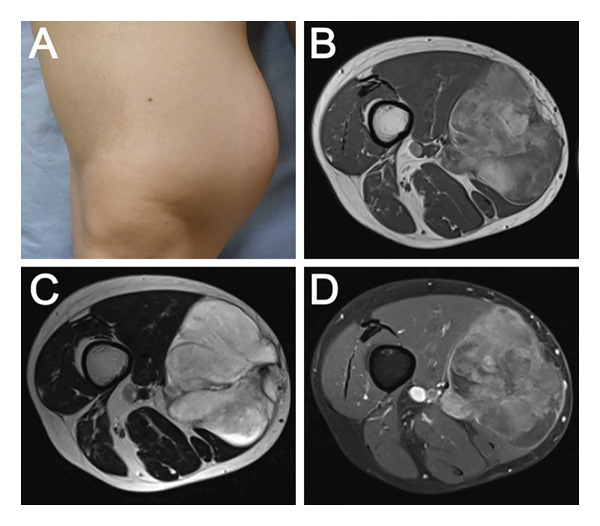
Findings on primary encounter. A mobile, elastic, and firm tumor (16 × 13 cm) was evident in the distal third of the right thigh (A). Axial MRI of the right thigh revealed a soft tissue mass inside the right sartorius. Compared to the surrounding muscle, the lesion was iso‐intense on T1‐weighted imaging (B). The lesion was hyperintense on T2‐weighted imaging, and there was no communication with the femoral vessels (C). The lesion was partially enhanced with gadolinium on T1‐weighted imaging with fat suppression (D). These findings suggested an extensive myxomatous tumor.

A 31‐year‐old man presented to a local orthopedic clinic with a three‐year history of asymptomatic swelling in the right thigh. The swelling had been progressively enlarging over the years, leading to his referral to our hospital for further evaluation and treatment. He had been in good health throughout his life and had never smoked. On physical examination, there was a mobile, elastic, and firm tumor measuring approximately 16 cm on the distal medial side of his right thigh, with no tenderness, pulsation, or Tinel‐like sign (Figure 1(A)). The white blood cell count was 6700/μL, with no evidence of neutropenia or lymphopenia. The C‐reactive protein (CRP) level was 0.02 mg/dL. Other laboratory findings were also within normal limits: hemoglobin, 16.0 g/dL; platelets, 31.8 × 10^4^/μL; albumin, 4.5 g/dL; aspartate aminotransferase, 30 U/L; alanine aminotransferase, 33 U/L; alkaline phosphatase, 64 U/L; lactate dehydrogenase, 214 U/L; creatinine, 0.89 mg/dL; and serum calcium, 9.1 mg/dL. The patient’s medical history was unremarkable. Specifically, there was no history of diabetes mellitus, immunosuppressive disorders, or the use of corticosteroids or biological agents. On physical reevaluation, the patient was entirely asymptomatic; specifically, no fatigue, weight loss, fever, night sweats, or respiratory symptoms such as cough or dyspnea were noted. Magnetic resonance imaging (MRI) revealed a soft tissue tumor located within the right sartorius muscle. The lesion was iso‐intense compared to the surrounding muscle on T1‐weighted images (Figure 1(B)), hyperintense on T2‐weighted images (Figure 1(C)), and heterogeneously enhanced with gadolinium, suggesting a myxomatous tumor (Figure 1(D)). The lesion was adjacent to the femoral vessels, but no invasion was apparent.

**FIGURE 2:**
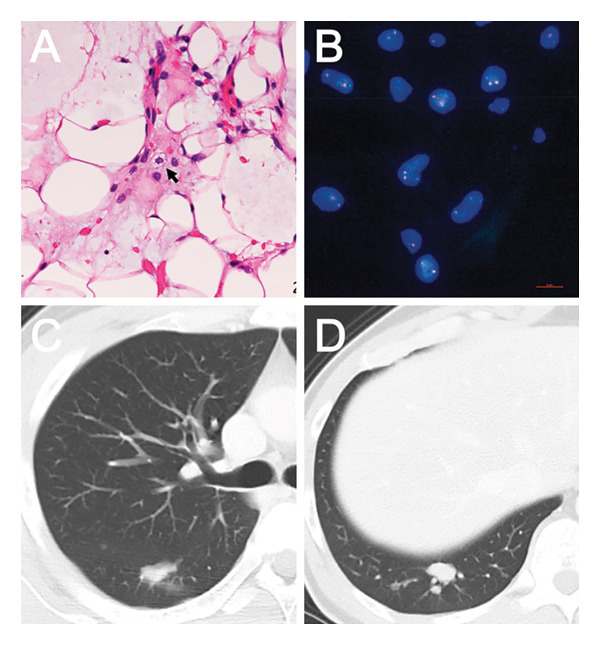
Pathological findings of the tumor revealed myxoid liposarcoma. On hematoxylin–eosin stain, lipoblasts (arrowhead) characterized the specimen along with atypical spindle cells and mature adipose‐like cells among abundant myxoid stroma (A). FISH assay revealed DDIT3 split signals in 74% (B). Staging whole‐body CT revealed pulmonary nodules in the right lung at S6 ((C) 20 × 9 mm) and S10 ((D) 17 × 10 mm). They were assumed to be pulmonary metastases, defining myxoid liposarcoma as Stage IV.

Since these findings were suggestive of a malignancy, an incisional biopsy was performed. The biopsy specimen showed atypical spindle cells scattered along with mature adipose‐like cells over an abundant myxoid stroma. Small numbers of lipoblasts were also seen on the specimen (Figure 2(A)). Immunohistochemical stains for MDM2, CDK4, and CD34 were negative, S‐100 protein was partially positive, and Ki‐67/MIB1 labeling index was approximately 10%. Fluorescence in situ hybridization (FISH) revealed DDIT3 split signals in 74% (Figure 2(B)). These findings indicated myxoid liposarcoma as the top differential diagnosis. The Fédération Nationale des Centers de Lutte Contre Le Cancer (FNCLCC) tumor grading [7] was Grade 1 (tumor differentiation: 2, mitotic count: 1, and tumor necrosis: 0). A pan‐scan computed tomography (CT) was performed to define the stage of the tumor. Two nodules, each 2 cm in diameter, were identified in the right lower lobe of the lung (S6 and S10) and were assumed to be metastatic lesions (Figures 2(C) and 2(D)). The tumor was diagnosed as myxoid liposarcoma AJCC Stage IV. Because the patient was young and healthy, the primary and metastatic lesions were presumed to be surgically resectable, and a multidisciplinary approach was selected.

**FIGURE 3:**
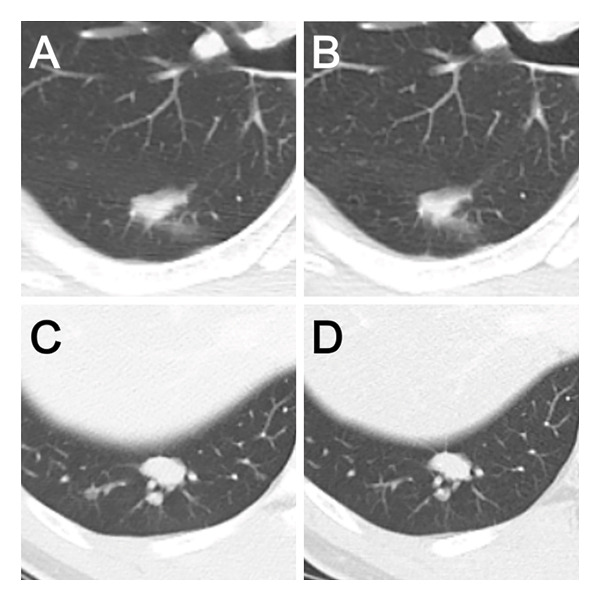
Clinical course of radiological findings. CT revealed two nodules in the right lower lobe at S6 (A) and S10 (C) before chemotherapy. The sizes of the two nodules remained unchanged after three courses of doxorubicin and ifosfamide (B) and (D).

Three courses of preoperative chemotherapy with doxorubicin and ifosfamide were administered. Follow‐up CT and MRI showed no change in the size of the tumor and lung nodules (Figure 3). The patient then underwent wide resection of the tumor, including the vastus medialis, sartorius, and gracilis muscles, leaving an intentional margin on the lateral side to avoid the femoral vessels (operation time: 2 h 24 min). Postoperatively, 65 Gy (RBE) of proton radiation therapy was administered. After confirming that no new metastases had appeared, a robot‐assisted thoracoscopic right lower lobectomy and lymph node dissection were performed to resect the two pulmonary lesions (operation time: 2 h 45 min).

**FIGURE 4:**
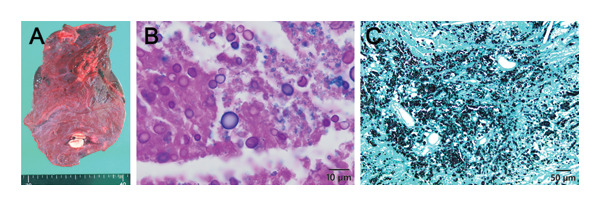
Right lower lobectomy revealed the diagnosis of pulmonary cryptococcosis. (A) Gross photograph of the resected right lower lobe. The specimen did not show any cavities or discoloration. (B) PAS‐Alcian blue stain. The capsules of the yeast cells are positively stained (inset), which is a characteristic finding of cryptococcus (scale bar = 10 μm). (C) GMS stain. Numerous round‐shaped yeasts are clearly highlighted in the nodules. No evidence of malignancy was seen.

Grossly, two white and solid nodules (18 and 17 mm in the largest diameter, respectively), as well as multiple miliary nodules, were found in the lung parenchyma. Histologic examination of the nodules, however, revealed no neoplastic lesions but rather necrotizing granulomas containing numerous round‐shaped encapsulated yeasts. The Grocott’s methenamine silver (GMS) stain and combined Alcian Blue–PAS stain were clearly positive, leading to a diagnosis of pulmonary cryptococcosis (Figure 4). Pulmonary metastases were ruled out, and the tumor was restaged as myxoid liposarcoma, AJCC Stage Ib. Postoperative chemotherapy was canceled, and no local or distant recurrence has been observed over a three‐year follow‐up. The chronological summary of the patient’s clinical course, including the timing of treatments and the eventual diagnostic revision, is presented in Table 1.

**TABLE 1:** Chronological timeline of clinical course and treatment.

Time point	Clinical event/intervention	Diagnostic status
3 years before referral	Patient noticed asymptomatic swelling in the right thigh	Observation

Month 0 (initial presentation)	MRI, incisional biopsy, and staging CT +2	Myxoid liposarcoma, Stage IV (presumed lung metastasis)

Months 1–3	Three courses of preoperative chemotherapy (AI therapy)	Stable disease

Month 4	Wide resection of the primary thigh tumor	Postoperative recovery

Months 5‐6	Proton radiation therapy (65 Gy [RBE])	Local control

Month 7	Robot‐assisted thoracoscopic right lower lobectomy	Revised diagnosis: Stage Ib (confirmed pulmonary cryptococcosis)

Month 8	Postoperative chemotherapy was canceled	Shift to observation

3‐year follow‐up	Final evaluation	No recurrence

## 3. Discussion

We report a case of pulmonary cryptococcosis mimicking lung metastasis in a patient with myxoid liposarcoma. After resection of the lung lesion, the AJCC stage was revised from Stage IV to Stage Ib when malignancy was ruled out. This reclassification was possible only because the “metastases” were presumed to be surgically resectable, and a multidisciplinary approach was taken.

Pulmonary cryptococcosis is an invasive lung mycosis caused by *Cryptococcus neoformans* or *Cryptococcus gattii*. The pathogen forms cavitary or nodular lesions in the lungs and can present with nonspecific symptoms such as cough, dyspnea, chest pain, or fever. However, it may also be completely asymptomatic. Risk factors for pulmonary cryptococcosis include immunodeficiency, immunosuppressive therapy, malignancy, diabetes, and cirrhosis [8]. Although pulmonary cryptococcus usually occurs in immunocompromised patients, there is an emerging trend in immunocompetent patients [9]. Half of immunocompetent patients are asymptomatic, which is why the disease is underdiagnosed [9, 10]. In addition, there are no characteristic radiographic features of pulmonary cryptococcosis. It may present as single or multiple nodules, segmental consolidation, cavitation, bronchopneumonia, or mass‐like appearances and is usually located in the peripheral area of the lung. Due to its various radiographic manifestations, it can mimic lung cancer, pulmonary tuberculosis, bacterial pneumonia, or other pulmonary mycoses [10, 11]. In our case, the patient had been aware of a tumorous lesion in the thigh for 3 years, and given the long duration, the lung lesion was initially considered a metastatic finding. However, pathological diagnosis revealed it to be pulmonary cryptococcosis. This case highlights the importance of considering pulmonary cryptococcosis in the differential diagnosis, even when a metastatic lesion is suspected.

Although rare, several studies have documented the coexistence of pulmonary cryptococcosis and sarcomas, primarily in pediatric and young adult populations [12, 13]. Allende et al. reported four cases (ages 8–16 years) of soft tissue and bone sarcomas (Ewing sarcoma and rhabdomyosarcoma) where asymptomatic pulmonary nodules were detected during routine follow‐up while in remission [12]. In those cases, the nodules appeared after intensive multiagent chemotherapy—specifically including vincristine, doxorubicin, cyclophosphamide, and ifosfamide—and, in three patients, total body irradiation. The authors suggested that such treatments could impair cell‐mediated immunity, thereby increasing the risk of cryptococcal infection. Similarly, Sakamoto and Hisaoka reported a 22‐year‐old patient with Ewing sarcoma who developed a solitary pulmonary nodule following the completion of a multiagent chemotherapy regimen [13].

Common to all previously reported cases was the initial clinical assumption that these new nodules represented metastatic disease, which led to surgical resection. A critical distinction in our case is the timing of the presentation. While previous reports described nodules appearing during or after treatment in patients otherwise in remission, our patient presented with pulmonary lesions at the time of the primary tumor diagnosis, before any immunosuppressive therapy. This led to an initial misclassification as Stage IV disease. These findings underscore that pulmonary cryptococcosis should be considered in the differential diagnosis of lung nodules in sarcoma patients not only postchemotherapy but also at initial presentation, regardless of the apparent immune status.

Due to its various radiographic manifestations, pulmonary cryptococcosis can closely mimic lung cancer or metastatic disease. A recent report by Ohnishi et al. further illustrates this diagnostic challenge, describing a case that radiologically mimicked lung cancer with multiple pulmonary metastases [14]. They noted that more than 40% of patients with histopathologically diagnosed pulmonary cryptococcosis were initially misdiagnosed, highlighting that radiological evidence alone is often insufficient for differentiation.

Reflecting on these diagnostic pitfalls, a significant limitation in the management of our case was the omission of less invasive microbiological evaluations prior to surgical intervention. Specifically, serum cryptococcal antigen testing, β‐D‐glucan levels, and sputum cultures were not obtained because the clinical focus was primarily on treating presumed metastatic sarcoma. While histopathological examination with GMS and Alcian Blue–PAS stains ultimately provided a definitive diagnosis, our experience underscores that clinicians should consider noninvasive fungal screening, particularly when pulmonary nodules show a poor response to systemic chemotherapy, to avoid potentially unnecessary or overly invasive procedures.

## 4. Conclusion

We experienced a case of pulmonary cryptococcosis that was initially presumed to be pulmonary metastasis of myxoid liposarcoma at diagnosis. By surgically resecting all lesions under the assumption that they were resectable, metastasis was ruled out, leading to a modification of the tumor stage. Although rare, this case reemphasized the importance of establishing preoperative pathological diagnoses, even for lesions presumed to be “metastatic” in soft tissue sarcoma.

## Author Contributions

Masatake Matsuoka was involved in the design of the study, performed the clinical assessment and analysis and interpretation of data, and drafted and revised the manuscript. Mariko M. Kawamura, Hideki Ujiie, Shin Ariga, Shinichi Nakazato, and Kenjiro Kato were involved in the design of the study, assisted with data interpretation, and revised the manuscript for important intellectual content. Takayuki Hashimoto, Yoshihiro Matsuno, Tatsuya Kato, and Norimasa Iwasaki were involved in the design of the study and data acquisition and revised the manuscript critically for important intellectual content.

## Funding

This research received no specific grant from any funding agency in the public, commercial, or not‐for‐profit sectors.

## Disclosure

All authors have read and approved the final manuscript.

## Ethics Statement

The authors declare that appropriate written informed consent was obtained from the patient for the publication of this case report and accompanying images.

Institutional review board approval is typically not required for a single case report at our institution, but the study was conducted with the approval of the relevant department heads.

## Consent

Please see the Ethics Statement.

## Conflicts of Interest

The authors declare no conflicts of interest.

## Data Availability

The data that support the findings of this study are available from the corresponding author upon reasonable request.

## References

[1] Bovée J. , Bloem J. , Flanagan A. , Nielsen G. , and Yoshida A. , WHO clas-sification of Tumours Editorial Board, Soft Tissue and Bone Tumours Lyon (France): International Agency for Research on Cancer. (2020) .

[2] Homsy P. , Bohling T. , Seitsonen A. , Sampo M. , Tukiainen E. , and Blomqvist C. , Patterns of Metastatic Recurrence of Genetically Confirmed Myxoid Liposarcoma, Annals of Surgical Oncology. (2023) 30, no. 7, 4489–4497, 10.1245/s10434-023-13312-x. 36907960 PMC10250512

[3] Blay J. Y. , Toulmonde M. , Valentin T. et al., Clinical Presentation, Management and Outcome of 11,132 Patients with Liposarcoma Patients: a Population-based Study from the NETSARC+ Registry, Lancet Regression Health European. (2025) 57, 10.1016/j.lanepe.2025.101403. PMC12336835 40799506

[4] Casali P. G. , Abecassis N. , Aro H. T. et al., Soft Tissue and Visceral Sarcomas: ESMO-EURACAN Clinical Practice Guidelines for Diagnosis, Treatment and follow-up, Annals of Oncology. (2018) 29, no. Suppl 4, iv51–iv67, 10.1093/annonc/mdy096, 2-s2.0-85052604766. 29846498

[5] Kawai A. , Araki N. , Ae K. et al., Japanese Orthopaedic Association (JOA) Clinical Practice Guidelines on the Management of Soft Tissue Tumors 2020-Secondary Publication, Journal of Orthopaedic Science. (2022) 27, no. 3, 533–550, 10.1016/j.jos.2021.11.023. 35339316

[6] Amin M. B. , Greene F. L. , Edge S. B. et al., The Eighth Edition AJCC Cancer Staging Manual: Continuing to Build a Bridge from a Population-Based to a More “Personalized” Approach to Cancer Staging, CA: A Cancer Journal for Clinicians. (2017) 67, no. 2, 93–99, 10.3322/caac.21388, 2-s2.0-85009446471. 28094848

[7] Trojani M. , Contesso G. , Coindre J. M. et al., Soft-Tissue Sarcomas of Adults; Study of Pathological Prognostic Variables and Definition of a Histopathological Grading System, International Journal of Cancer. (1984) 33, no. 1, 37–42, 10.1002/ijc.2910330108, 2-s2.0-0021366779. 6693192

[8] Chang C. C. , Sorrell T. C. , and Chen S. C. , Pulmonary Cryptococcosis, Seminars in Respiratory and Critical Care Medicine. (2015) 36, no. 5, 681–691, 10.1055/s-0035-1562895, 2-s2.0-84942240708. 26398535

[9] Zhang Y. , Li N. , Zhang Y. et al., Clinical Analysis of 76 Patients Pathologically Diagnosed with Pulmonary Cryptococcosis, European Respiratory Journal. (2012) 40, no. 5, 1191–1200, 10.1183/09031936.00168011, 2-s2.0-84868575765. 22408204

[10] Setianingrum F. , Rautemaa-Richardson R. , and Denning D. W. , Pulmonary Cryptococcosis: a Review of Pathobiology and Clinical Aspects, Medical Mycology. (2019) 57, no. 2, 133–150, 10.1093/mmy/myy086, 2-s2.0-85059261215. 30329097

[11] Kontoyiannis D. P. , Peitsch W. K. , Reddy B. T. et al., Cryptococcosis in Patients with Cancer, Clinical Infectious Diseases. (2001) 32, no. 11, E145–E150, 10.1086/320524, 2-s2.0-0942298263. 11340547

[12] Allende M. , Pizzo P. A. , Horowitz M. , Pass H. I. , and Walsh T. J. , Pulmonary Cryptococcosis Presenting as Metastases in Children with Sarcomas, The Pediatric Infectious Disease Journal. (1993) 12, no. 3, 240–243, 10.1097/00006454-199303000-00014, 2-s2.0-0027416915. 8451102

[13] Sakamoto A. and Hisaoka M. , Pulmonary Cryptococcosis Mimicking a Metastasis in a Patient with Ewing Sarcoma, Respirology Case Reports. (2016) 4, no. 5, 10.1002/rcr2.181, 2-s2.0-85066152450. PMC5256956 28127434

[14] Ohnishi H. , Horino T. , Nakajima H. , and Yokoyama A. , Pulmonary Cryptococcosis Mimicking Lung Cancer with Multiple Lung Metastases, Respirology Case Reports. (2024) 12, no. 6, 10.1002/rcr2.1415. PMC11175837 38872912

